# A Segmentation Method for Lung Parenchyma Image Sequences Based on Superpixels and a Self-Generating Neural Forest

**DOI:** 10.1371/journal.pone.0160556

**Published:** 2016-08-17

**Authors:** Xiaolei Liao, Juanjuan Zhao, Cheng Jiao, Lei Lei, Yan Qiang, Qiang Cui

**Affiliations:** 1 College of Computer Science and Technology, Taiyuan University of Technology, Taiyuan, 030024, China; 2 PET/CT center of Shanxi coal Central Hospital, Taiyuan, Shanxi, 030024, China; Beijing University of Technology, CHINA

## Abstract

**Background:**

Lung parenchyma segmentation is often performed as an important pre-processing step in the computer-aided diagnosis of lung nodules based on CT image sequences. However, existing lung parenchyma image segmentation methods cannot fully segment all lung parenchyma images and have a slow processing speed, particularly for images in the top and bottom of the lung and the images that contain lung nodules.

**Method:**

Our proposed method first uses the position of the lung parenchyma image features to obtain lung parenchyma ROI image sequences. A gradient and sequential linear iterative clustering algorithm (GSLIC) for sequence image segmentation is then proposed to segment the ROI image sequences and obtain superpixel samples. The SGNF, which is optimized by a genetic algorithm (GA), is then utilized for superpixel clustering. Finally, the grey and geometric features of the superpixel samples are used to identify and segment all of the lung parenchyma image sequences.

**Results:**

Our proposed method achieves higher segmentation precision and greater accuracy in less time. It has an average processing time of 42.21 seconds for each dataset and an average volume pixel overlap ratio of 92.22 ± 4.02% for four types of lung parenchyma image sequences.

## Introduction

Lung cancer is one of the most common causes of cancer-related death worldwide [1]. Computed tomography (CT) [2] scanning technology has good density resolution for lesions in the human body and is currently the most effective and direct imaging method for the early diagnosis of lung cancer. However, as the accuracy requirements for clinical imaging of lesions increase, the CT scanning thickness decreases, and a large number of CT image sequences need to be produced [3]. The massive amount of image data will inevitably increase the challenge of CT image processing, leading to a slow processing speed and decreased efficiency. In addition, because each pulmonary CT image presents a different morphological structure from the top to the bottom of the lung in CT image sequences, the general segmentation algorithm is not effective. Therefore, determining how to segment the lung parenchyma image sequences quickly without reducing accuracy is of great significance for the subsequent segmentation of pulmonary nodules and benign and malignant diagnoses.

Lung segmentation can be an important component of computer-aided diagnosis (CAD) systems [4]. Geng H’s group used an iterative gray threshold to select seed points automatically and then extract each lung parenchyma image with the region growing method, which is sensitive to background noise [5]. Liming D and colleague present a new form of lung parenchyma segmentation. The optimal threshold value method and the boundary tracking method are used to segment the lung region and can effectively eliminate the influence of background noise but may lose some of the lung parenchyma [6]. Mansoor A and coworkers segmented the lung parenchyma in two steps [7] by using the fuzzy connectedness (FC) image segmentation algorithm to perform the initial lung parenchyma extraction and then texture-based local descriptors to segment abnormal imaging patterns using a near-optimal keypoint analysis. However, this method is not effective for processing irregular images. Wavelet transform has been applied by Shojaii R [8] to decompose an image into several regions, and the regions with low pixel intensities are kept and grown to segment the honeycomb regions. This method can effectively segment irregular lung parenchyma images. Yan-hua and coworkers used several methods, including the optimal iterative threshold, three-dimensional connectivity labeling, and three-dimensional region growing methods, for the initial segmentation of the lung parenchyma and used the morphological method to repair the lung parenchyma [9]. Luo X [10] and others used an improved active contour model, which can obtain better segmentation result with the help of artificial segmentation but is very time consuming. There are also some scholars who use superpixel to segment medical images. Yu N and Weinstein S P [11] proposed a novel automatic segmentation framework for tumor on breast DCE-MRI images by using graph-cuts and superpixel classification, which can achieve a classification accuracy of 96%.

Superpixel was also used for bacteria cell segmentation by Song Y’s group [12]. Features of superpixels are extracted and trained by supervised deep learning method with an accuracy of 99% and a sensitivity of 100% for four types of different bacteria.

In general, lung segmentation methods are based on threshold, region, and mathematical morphology. However, lung CT images are sequential, and the existing methods in the study of lung parenchyma segmentation algorithms are generally for single-image segmentation of CT images and ignore the before-to-after image correlation. A few scholars have studied sequential image segmentation, but this often involves a long processing time, low efficiency and extensibility.

In this paper, we use the position particularity of the lung parenchyma in lung CT images, fully consider the strong correlation between adjacent slices of CT image sequences, and put forward a segmentation method for lung parenchyma image sequences based on superpixels and a self-generating neural forest. The experimental results show that our proposed method can significantly increase the speed of segmentation for four types of lung parenchyma images, which guarantees accuracy and integrity.

## Materials and Methods

### 2.1 Materials

#### 2.1.1 Ethics statement

This study was approved by the institutional review board (IRB) of the Coal Center Hospital in Shanxi. The study was conducted in accordance with the hospital’s ethics requirements. Informed consent was obtained from all patients for being included in the study.

#### 2.1.2 Datasets

The CT image datasets used in this study were obtained from a hospital in Shanxi Province, China. All data can be accessed at https://figshare.com/s/254e3467efd57a442334. We used a Discovery ST16 PET-CT scanner from the General Electric Company of America (150 mA, 140 kV, with a slice thickness of 3.75 mm). In the experiment, we select lung CT sequence image datasets from 80 people with a total of 4812 CT images, and the size of each image was 512 × 512. Based on the physician’s prior knowledge and the morphological perspective of lung CT image sequences, the 80 datasets were divided into four categories: without nodules, benign nodules, malignant SPN (solitary pulmonary nodules) and pleural nodules. Each category had 20 datasets and approximately 1200 CT images.

### 2.2 Proposed Method

**Fig 1:**
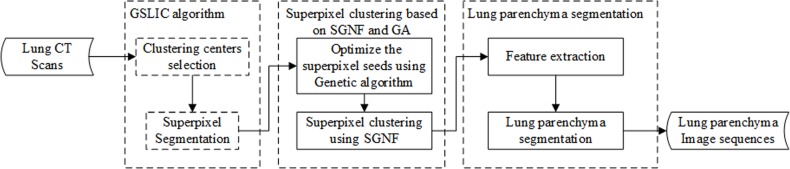
Diagram of the lung parenchyma image sequences segmentation.

We propose a segmentation method for lung parenchyma image sequences based on superpixels and a self-generating neural forest that mainly involves a gradient and sequential linear iterative clustering algorithm (GSLIC) to obtain superpixels, clustering of superpixels with a self-generating neural forest (SGNF), and lung parenchyma image sequences segmentation. A block diagram of the lung parenchyma image sequences segmentation is shown in **Fig 1**.

#### 2.2.1 Gradient and sequential linear iterative clustering (GSLIC)

It has been difficult to obtain better segmentation in a relatively short time in lung parenchyma image sequence segmentation. To solve this problem, we used multiple CT images and drew on the prior knowledge of the physicians to propose a superpixel segmentation algorithm for image sequence. Our method is based on a gradient and sequential linear iterative clustering (GSLIC) algorithm and includes lung ROI image sequence extraction and ROI sequence superpixel segmentation.

**Fig 2:**
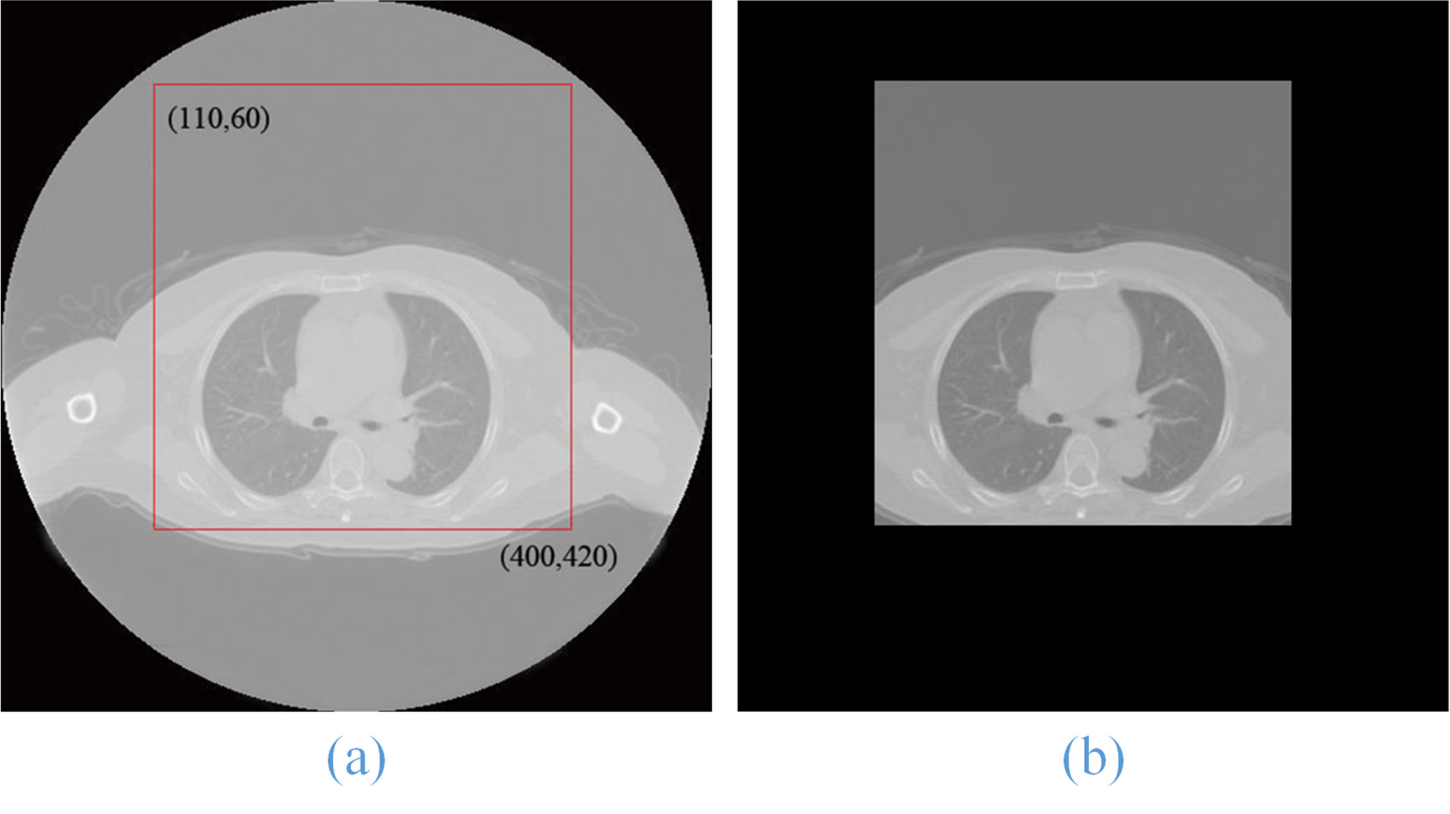
Lung ROI extraction result (b) of the original lung CT image (a).

1. Lung ROI sequence extraction: Because the position of the lung parenchyma region in CT images is relatively fixed, we increased the running speed by first extracting the lung ROI sequences using a statistical method for ROI extraction that is adopted in this paper. More than 4800 CT lung images of 80 individuals were analyzed, and we determined that the rectangles in the upper-left (100, 60) and lower-right corners (400,420) could include all lung parenchyma regions. Therefore, we can obtain the entire lung ROI sequence based on the two points in the CT image sequence. In **Fig 2**, the original lung CT image **(a)** was used to extract its ROI image **(b)**. Extracting the lung ROI sequences can reduce the processing time and simultaneously eliminate some noise.

2. Superpixel segmentation on ROI image sequences: The concept of superpixels was first put forward by Ren [13] in 2003. A superpixel is a collection of pixels with similar characteristics, such as color, brightness, and texture. An image can be composed of a certain number of superpixels that contain multiple combination characteristics of the pixels and can preserve the edge information of the original image. Compared with a single pixel, a superpixel contains rich characteristic information and can greatly reduce image post-processing complexity and significantly increase the speed of image segmentation.

Traditional superpixel segmentation uses a process of simple, linear iterative clustering. After this method was improved by Lucchi, Hammoudi, and Wang J [14–16], it was applied to a single image segmentation. In this paper, an algorithm based on gradient and sequential linear iterative clustering (GSLIC) is proposed to segment image sequences. Each pixel in the sequence of lung CT images can be represented by a six-dimensional feature vector (**[*l*, *a*, *b*, *x*, *y*, *z*]** ^**T**^). The similarity between the pixels can be measured by the *Euclidean* distance between them. A pixel’s feature vector is made up of its color vector **[*l*, *a*, *b*]** in ***CIELAB*** color space and its space coordinate vector **[*x*, *y*, *z*]**, where ***x*** and ***y*** are the pixel coordinates, and ***z*** is the serial number of the image. The **GSLIC** procedure is shown in **Table 1**.

**Table 1:** GSLIC algorithm.

*Algorithm 1 GSLIC Algorithm*
1: Initialize cluster centers ***C***_***k***_ **= [*L***_***k***_**, *a***_***k***_**, *b***_***k***_**, *x***_***k***_**, *y***_***k***_**, *z***_***k***_**]**^***T***^ by sampling pixels at regular grid steps.
2: Choose cluster centers in an ***n*** * ***n*** neighborhood, to the lowest gradient position.
3: ***Repeat***
4: ***for*** each cluster center ***C***_***k***_ in ***z***_***k***_ ***do***
5: Assign the best matching pixels from an ***2S*** * ***2S*** square neighborhood around the cluster center according to the distance measure.
6: ***end for***
7: Compute new cluster centers and residual error ***E***{distance between previous centers and recomputed centers}.
8: ***until E*** < = threshold. Save cluster centers ***C***_***k***_ and transmit ***C***_***k***_ to the next image ***z***_***k+1***_.
9: ***until*** all image sequences are segmented.
10: Enforce connectivity.

In an original CT image with ***N*** pixels that needs to be divided into ***K*** superpixels, each superpixel contains approximately ***N*/*K*** pixels, and therefore, the average length of each superpixel ***S*** is about (1).

$$S=\sqrt{\left(N/K\right)}$$ (1)

We first take an initial clustering center every ***S*** pixels and then select cluster centers using a 3 * 3 nuclear window to the lowest gradient position. When selecting the initial clustering center, a method of gradient descent is adopted to sample pixels at a regular grid so that the edge points are not selected as the cluster centers. By using a *3* * *3* nuclear window, a pixel’s gradient *G*(*x*, *y*) can be defined as (2).

$$G(x,y)={\left[V(x+1,y)-V(x-1,y)\right]}^{2}+{\left[V(x,y+1)-V(x,y-1)\right]}^{2}$$ (2)

The ***Min* {*G*(*x*, *y*)}** coordinates in each grid can be chose as the cluster center. Following that, each clustering center can be search for neighboring similar pixels around the search space for ***2S*** * ***2S*** based on the similarity of ***Ds*** between the pixels.

In our method, for the same CT image with serial number ***z***, the similarity of ***Ds*** between the pixels (**[*l***_***j***_**, *a***_***j***_**, *b***_***j***_**, *x***_***j***_**, *y***_***j***_**, *z*]** ^**T**^) to the clustering center (**[*l***_***i***_**, *a***_***i***_**, *b***_***i***_**, *x***_***i***_**, *y***_***i***_**, *z*]** ^**T**^) can be calculated by their color feature distance ***D***_***lab***_ and space feature distance ***D***_***xy***_. The calculation formulas of ***D***_***lab***_, ***D***_***xy***_ and ***Ds*** are as follows in (3), (4) and (5).

$$D_{lab}=\sqrt{{\left(l_{j}-l_{i}\right)}^{2}+{\left(a_{j}-a_{i}\right)}^{2}+{\left(b_{j}-b_{i}\right)}^{2}}$$ (3)

$$D_{xy}=\sqrt{{\left(x_{j}-x_{i}\right)}^{2}+{\left(y_{j}-y_{i}\right)}^{2}}$$ (4)

$$Ds=\frac{D_{lab}+\delta D_{xy}}{\sqrt{1+\delta^{2}}}$$ (5)

**Fig 3:**
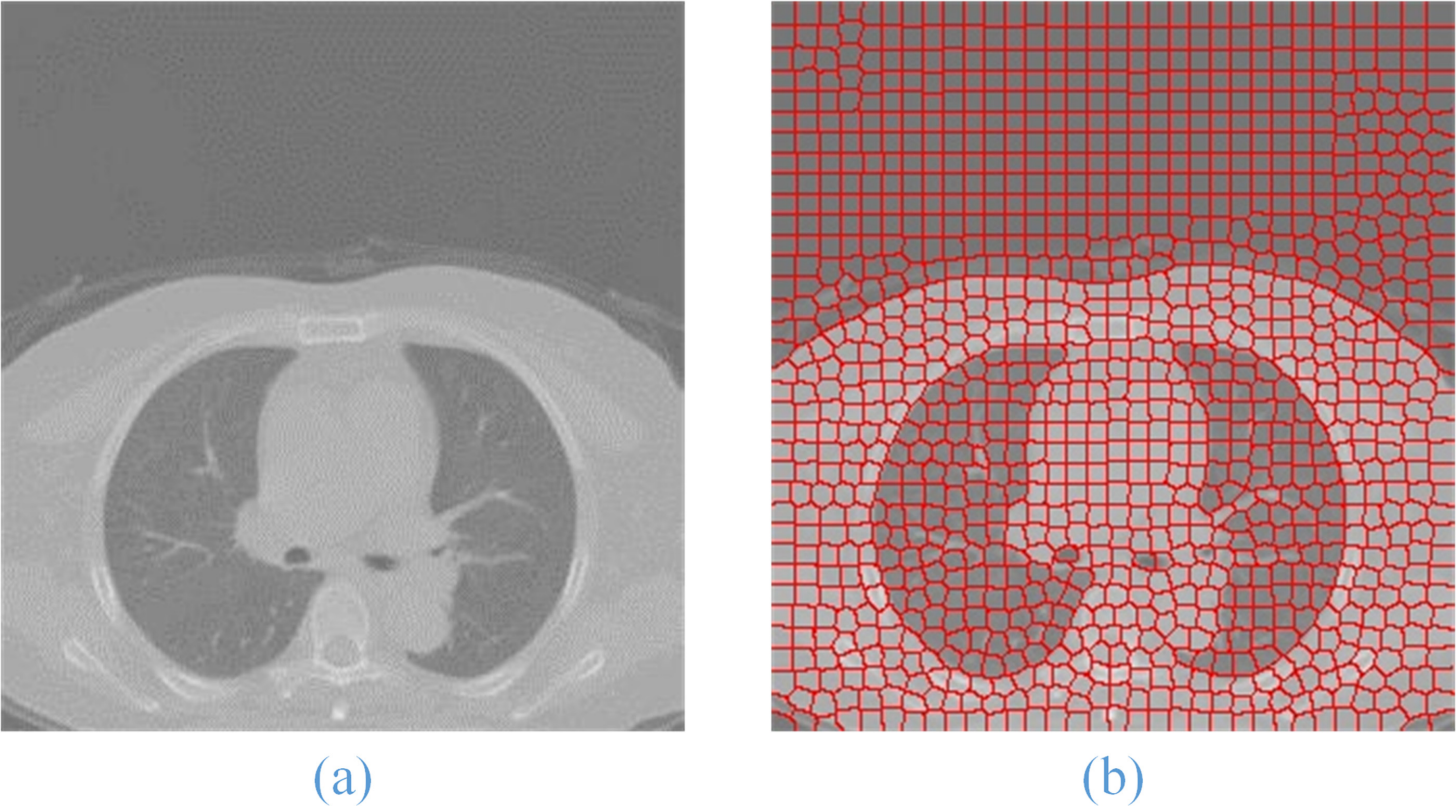
Superpixel segmentation result (b) of the original lung ROI image (a).

In (5), ***δ*** is a parameter to adjust the weight of ***D***_***lab***_ and ***D***_***xy***_. The larger the value, the bigger the weight of ***D***_***xy***_ to calculate ***Ds*** will be, which is generally between 1 and 20. The result of superpixels segmentation on ROI image is shown in **Fig 3.**

The proposed method can segment an image into a series of superpixels, each of which can also be expressed by feature vector (**[*l*, *a*, *b*, *x*, *y*, *z*]** ^**T**^). In the process of obtaining superpixels, effective clustering centers can be chose by gradient descent, and the blocks can be obtained through the clustering algorithm. In addition, taking the correlation between the sequences of CT images into account, the coordinate information of the clustering centers in the previous image is directly transmitted to the next image, which can significantly improve the image’s superpixel segmentation speed.

#### 2.2.2 Superpixel clustering based on SGNF and GA

In this paper, a method using a self-generated neural forest (SGNF) algorithm optimized by a genetic algorithm (GA) was proposed to cluster the superpixels. The genetic algorithm is used to select the optimal clustering centers, which are used to generate the neural trees that form the neural forest. Our method effectively overcomes the instability of the primary SGNN and improves the efficiency and accuracy of clustering.

1. Self-generating neural tree (SGNT): Self-generated neural networks (SGNNs) were developed in 1992 [17] using a competitive learning mechanism for samples learning. A self-generating neural tree (SGNT) is generated by an SGNN using unsupervised learning.

An SGNT includes neurons, weights, and connections. In this paper, we use an ordered pair < {*n*_*j*_}, {*l*_*k*_} > to express an SGNT, where {*n*_*j*_} is the set of neurons, and {*l*_*k*_} is the set of connections. Each neuron also can be represented as ordered pair < *w*, {*n*_*c*}_>, where ***w*** is the weight of the neuron, and {*n*_*c*_} is the set of child neurons of the neuron. Each leaf neuron corresponds to a sample, and each root neuron is a cluster center. All leaf neurons of the root neuron belong to the same cluster, and the weight of every neuron is the average attribute of all the leaf neurons it covers.

**Fig 4:**
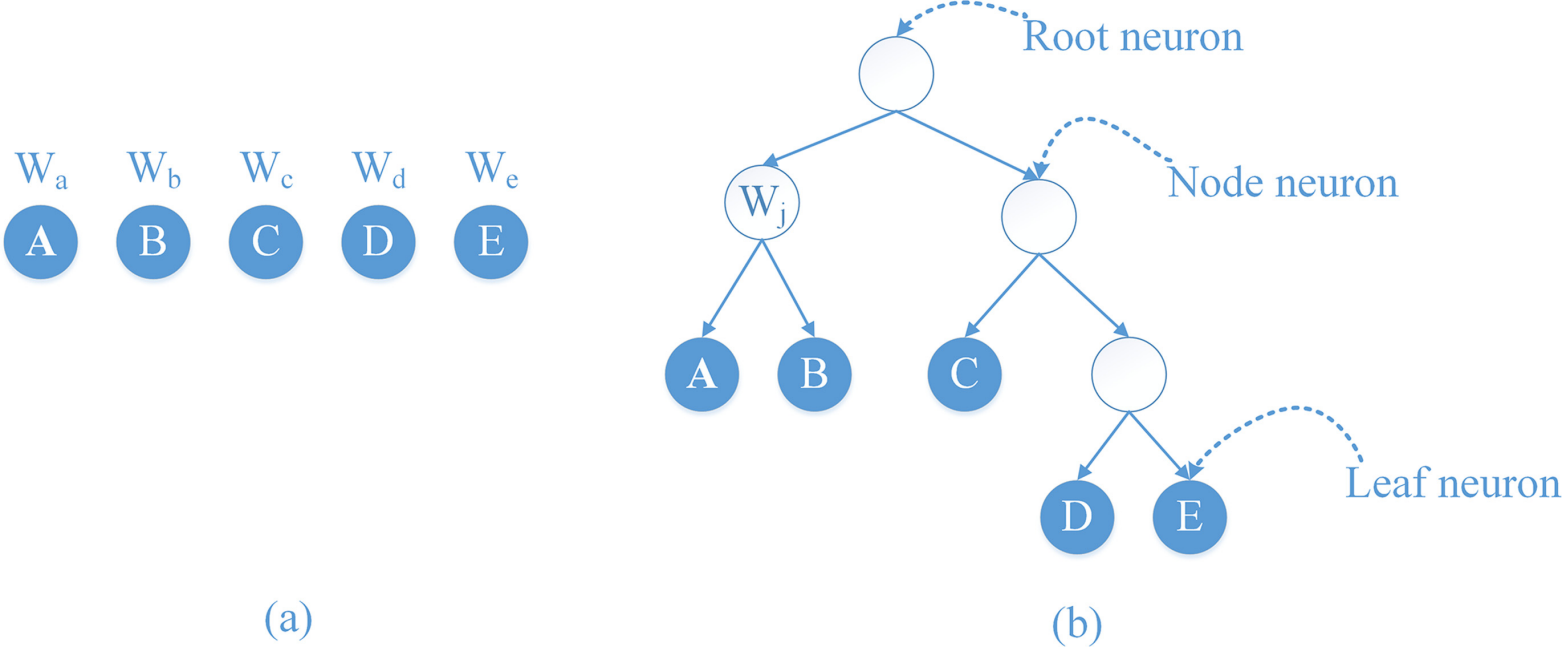
The structure of the SGNT (b) generated by five samples (a).

Therefore, the structure of an SGNN shows simplicity and a good self-organizing capability and learning speed. It is beneficial to learn clustering that has high performance. **Fig 4** shows the structure of an SGNT with five samples. **Fig 4(A)** lists a clustering sample set where *W*_*j*_, j = a, b, …, e are the sample attributes. **Fig 4(B)** is the SGNT generated by following SGNT generating rules [18–20].

2. Self-generating neural forest (SGNF) optimized by GA: Despite its good capacity for clustering, an SGNT is influenced by the input order of the samples [21]. To solve this problem, we propose an adaptive clustering algorithm that is optimized by GA and in which the SGNT is generalized to a self-generating neural forest (SGNF), and the GA is applied to select optimal superpixel seeds as the initial input into the SGNF. The process of clustering with an SGNF optimized by a GA is shown in **Table 2**.

**Table 2:** SGNF optimized by GA.

*Algorithm 2 SGNF Optimized by GA*
1: Input the initial sample set **{*X*}**, and the ***K*** initial seeds are selected randomly.
2: Obtain the optimal ***K*** superpixel seeds to generate the initial ***SNGF*** with ***K*** neural tree using genetic algorithm.
3: ***Repeat***
4: ***For*** each superpixel sample in **{*X*}** do
5: Generate neuron ***n***_***j***_ from **{X}** and traverse the SGNF to find a neuron ***n***_***win***_ with the shortest distance to ***n***_***j***_.
6: Connect ***n***_***j***_ to the ***SGNT***.
***If n***_***win***_ is a leaf neuron in the current SGNT
Create a new neuron ***n***_***j+1***_ and copy weight ***w***_***win***_ to ***w***_***j+1***_;
Connect ***n***_***j***_ and ***n***_***j+1***_ to ***n***_***win***_ as a child.
***Else*** Connect ***n***_***j***_ to ***n***_***win***_ as a child, update node weight.
7: ***End for*.**
8: ***Until*** all samples have been input into the ***SGNF***_._

**Fig 5:**
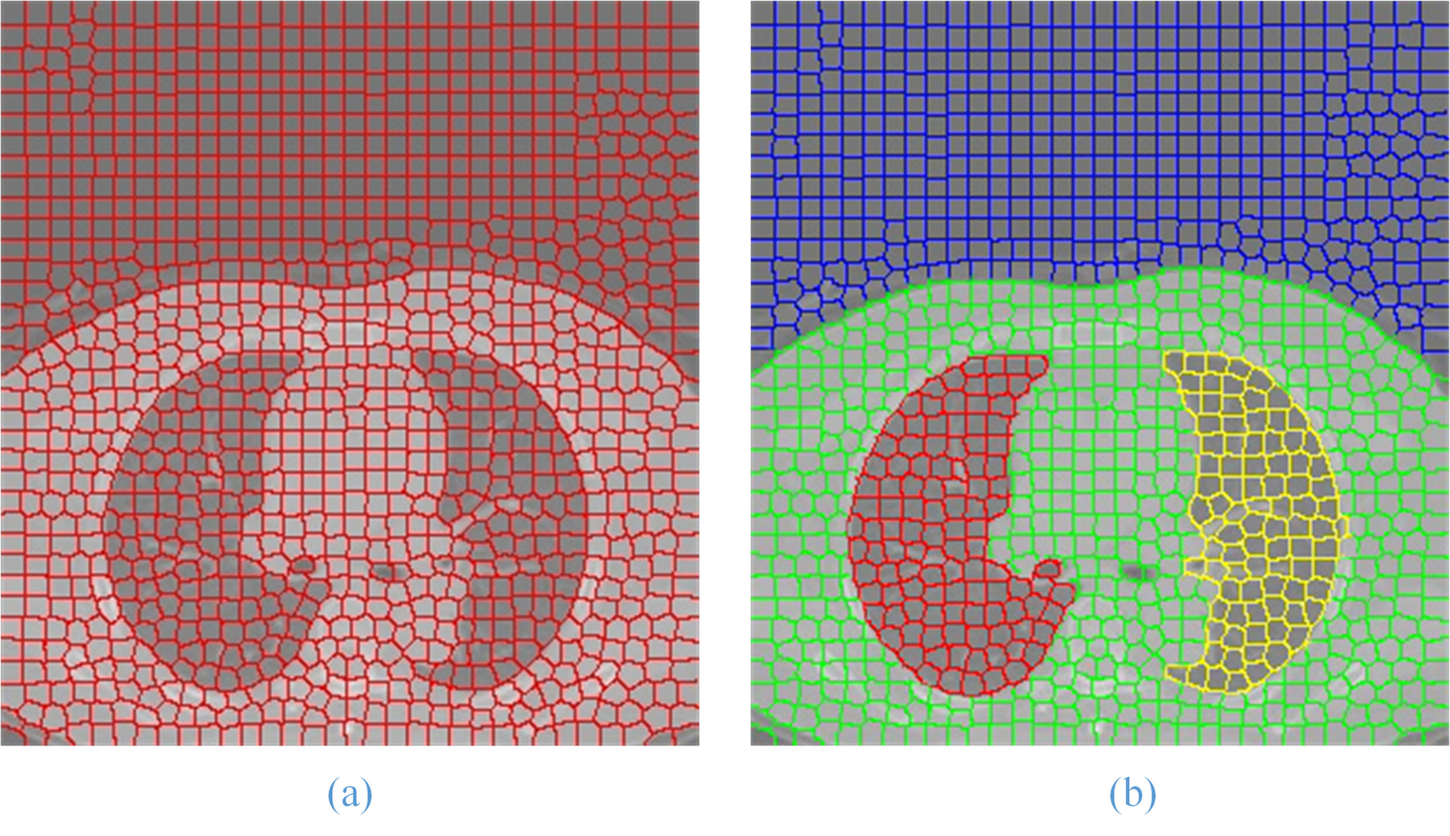
Superpixel clustering result (b) of the superpixel segmentation image (a).

Each SGNT in an SGNF corresponds to a cluster, and all the leaf neurons in an SGNT belong to the same cluster. In **section 2.2.1**, we show that each superpixel can be expressed by a feature vector (**[*l*, *a*, *b*, *x*, *y*, *z*]** ^**T**^). For given sample {***X***_***i***_} where ***i*** = 1, 2, …, *L*, the distance between sample ***X***_***j***_ and clustering center ***X***_***i***_ can be calculated as (6):

$$\|X_{i},X_{j}\|=\sqrt{\frac{\sum_{k=1}^{p}{\left(w_{jk}-w_{ik}\right)}^{2}}{p}}$$ (6)

where ***k*** is the sequence number of the element in the feature vector, and w_*jk*_ is the weight of the first *k* attribute. When processing the superpixels segmented by GSLIC, the attributes of superpixels, such as color and coordinate feature values, can be used to generate an SGNF. The result of superpixel clustering with GA-SGNF is shown in **Fig 5**.

Different ***K*** seed samples generate a SGNF with different structures. Therefore, the choice of *K* seed samples can be seen as an optimization problem. To obtain preferable clustering results, the GA is used to search for *K* seed samples to optimize the clustering results as described in the next section.

3. Genetic Algorithm: The genetic algorithm (GA) is a method based on the probability search technology of population optimization [22–24]. The GA has a good ability for global searching and can search for the optimal solution quickly. In the previous section, each superpixel is expressed by a feature vector that can also correspond to the process of chromosome encoding in the GA. In addition, the sample capacity and clustering numbers are small, making the algorithm converge rapidly. Therefore, the optimal seed points will be found by the GA to obtain the best clustering results in a short time. The process of choosing optimal seed points using the GA is shown in **Table 3**.

**Table 3:** Genetic Algorithm.

*Algorithm 3 Genetic Algorithm*
1: Chromosome encoding.
2: Initialize the maximum iterations ***T*** and generate an initial population ***G (0)*** randomly.
3: Compute the fitness value φ(C) of each sample in initial population ***G (0)***.
4: ***If*** Δφ(C) < *μ* or iterations > T
5: Return to step 10.
6: ***Else*** perform genetic operators: selection, crossover and mutation.
7: Generate the next generation of population ***G (t+1)***.
8: Compute the fitness value φ(C) of each sample in initial population ***G (t+1)***.
9: Return to step 4.
10: Output the optimal solution ***C***_***opt***_.

We first define a chromosome structure **C** = **(*c***_***1***_**, *c***_***2***_**, *…*, *c***_***K***_**)**, where the c_i_, ***i*** = 1, 2, …, *K*, including initial *K* superpixels, and *K* is user-specified. For each superpixel with gene code string ***X***_***i***_ ***=* (*a***_***i1***_**, *a***_***i2***_**, *…*, *a***_***ip***_**)**, where in ***a***_***ip***_, ***i*** = 1, 2, …, *L*, ***p*** represents the number of superpixel attributes in **section 2.2.1**.

For the given superpixels {***X*:*X***_***1***_**,*X***_***2***_***…*,*X***_***L***_}, we finally obtain *K* classes of superpixels with cluster center R = (***r***_***1***_**, *r***_***2***_**, *…*, *r***_***K***_). The number of superpixels for cluster *r*_*i*_ is *n*_*i*_, and *x*_*ij*_ are all superpixels in cluster *r*_*i*_. We define a fitness function φ(C) that can be obtained by the between-class variance δ^2^ to evaluate the goodness of a chromosome. φ(C) and δ^2^ can be calculated using (7) and (8).

φ(C)=1(1+δ2) (7)

$$\delta^{2}=\sum_{i=1}^{K}\sum_{j=1}^{n_{i}}\sqrt{\frac{{\left\|x_{ij},r_{i}\right\|}^{2}}{n_{i}}}$$ (8)

The higher the value of φ(C), the better the chromosome quality is assumed to be. The chromosome ***C*** with the maximum value of φ(C) is considered the optimal one in the population, and the *K* superpixel seeds are chose to generate the SNGF.

The selection process copies individual strings with high fitness function values into the next population based on the ‘‘roulette wheel” selection approach. The main purpose of crossover is to exchange genetic information of the selected chromosomes. Mutation is the process of a random alteration in the genetic structure of a chromosome, which can introduce genetic diversity into the population. In our method, the probabilities of crossover and mutation are *τ* and *η*, respectively, and the termination criteria are as follows:

1. The biggest fitness function value is obtained and the algorithm converges.
2. The fixed number of generations is reached.

#### 2.2.3 Feature extraction and lung parenchyma segmentation

1. Feature extraction and lung identification: After clustering the superpixel samples using the optimized SGNF algorithm, four superpixel sample sets that include the left and the right lung parenchyma images, pleural tissue and extrathoracic area are obtained. We still need to identify and segment the lung parenchyma from the image sequences. Because the average greyscale value of each superpixel sample set is equal to the average value of all superpixels, the sample set with the highest value should be the pleural tissue, and the value for the lung parenchyma is close to that of the extrathoracic area. As the distribution of superpixel coordinates of the lung parenchyma is relatively concentrated, the sample set with the highest value of coordinate variance should be the extrathoracic area. And the left two sample sets should be the left and the right lung parenchyma. In this paper we will mainly extract two features of superpixel samples: the average grayscale value and the coordinate variance. An overview of the detection method is shown in **Table 4**.

**Table 4:** Feature extraction and lung parenchyma segmentation.

*Algorithm 4 Feature Extraction Algorithm*
1: Assume that the final four types of sample sets are {*S*_*i*_}, *i* = 1, 2, 3, 4, then
$\overset{4}{\underset{i=1}{\cup }}S_{i}=X,\;\underset{\left(1\leq i,j\leq 4,\;i\neq j\right)}{\forall }(S_{i}\cap S_{j})=\emptyset$
2: Calculate the average grayscale values *φ*(*S_i_*) for four sample sets using (9)
3: max(*φ*(*S_i_*)) → *pleural tissue*(*S*_4_)
4: Compute the centroid coordinates (*x_i_*,*y_i_*) and the coordinate variances *ξ*^2^(*S_i_*) of the three left sample sets {*S*_*i*_}using (10) and (11)
5: max(*ξ*^2^(*S_i_*)) → *extrathoracic area*(*S*_3_)
6: *two left sample sets* → *the left*(*S*_1_) *and the right*(*S*_2_) *lung* parenchyma
7: ***Traverse*** superpixel samples in **{***S*_*1*_, *S*_*2*_**}**
8: ***For*** *X_i_*,*X_j_* ∈ *S*_1_ ∪ *S*_2_, ***If*** *a*_*j6*_ = *a*_*i6*_, *X_i_*,*X_j_* → *N* (The same CT image ***N*** whose slice number is ***a***_***i6***_), Connect ***X***_***i***_ and ***X***_***j***_ to ***N***_***k***_.
9: ***Until*** all superpixel samples have been input.
10: ***Sequential output*** all ***N***_***k***_ corresponding to each lung parenchyma image.

As mentioned previously, each superpixel can be expressed by feature vector (**[*l*, *a*, *b*, *x*, *y*, *z*]** ^**T**^), ***X***_***i***_ ***=* (*a***_***i1***_**, *a***_***i2***_**, *…*, *a***_***i6***_**)**. For a sample set {**X**}, the formulas to determine the average grayscale value, centroid coordinate and coordinate variance are as follows:

$$\varphi (X)=\frac{1}{n_{i}}\sum_{i=1}^{n_{i}}\sqrt{a_{i1}^{2}+a_{i2}^{2}+a_{i3}^{2}}$$ (9)

$$\left(x_{0},y_{0})=\frac{1}{n_{i}}(\sum_{i=1}^{n_{i}}\sqrt{a_{i4}^{2}},\sum_{i=1}^{n_{i}}\sqrt{a_{i5}^{2}}\right)$$ (10)

$$\xi^{2}(X)=\frac{1}{n_{i}}\sum_{i=1}^{n_{i}}\sqrt{{\left(a_{i4}-x_{0}\right)}^{2}+{\left(a_{i5}-y_{0}\right)}^{2}}$$ (11)

The sample set with the smaller coordinate variance should be the left and the right lung parenchyma images, which are assumed to be S_1_ and S_2_. We still need to traverse all superpixel samples in S_1_ and S_2_. As the superpixels in the same image will have the same attribute value *z*, all superpixel samples can be connected according to the value of attribute *z*. By sequentially outputting all images, the sequential coarse lung parenchyma images were fully segmented.

2. Removing the trachea/bronchus and refining the lung contour: After coarse segmentation of lung CT image sequences, there are still the trachea/bronchus at the top of the lung image. To ensure the integrity of the lung parenchyma segmentation, we adopt an improved region growing method [25] to remove them. The description of improved region growing is given as follows.

**Step 1:** Binarization for the coarse lung image sequences
**Step 2:** Extract the minimum bounding rectangle of the lung.
**Step 3:** Select seed points by using LRS algorithm.
**Step 4:** Refine the lung contours with erosion and dilation.
**Step 5:** Acquire the final lung mask sequences.

**Fig 6:**
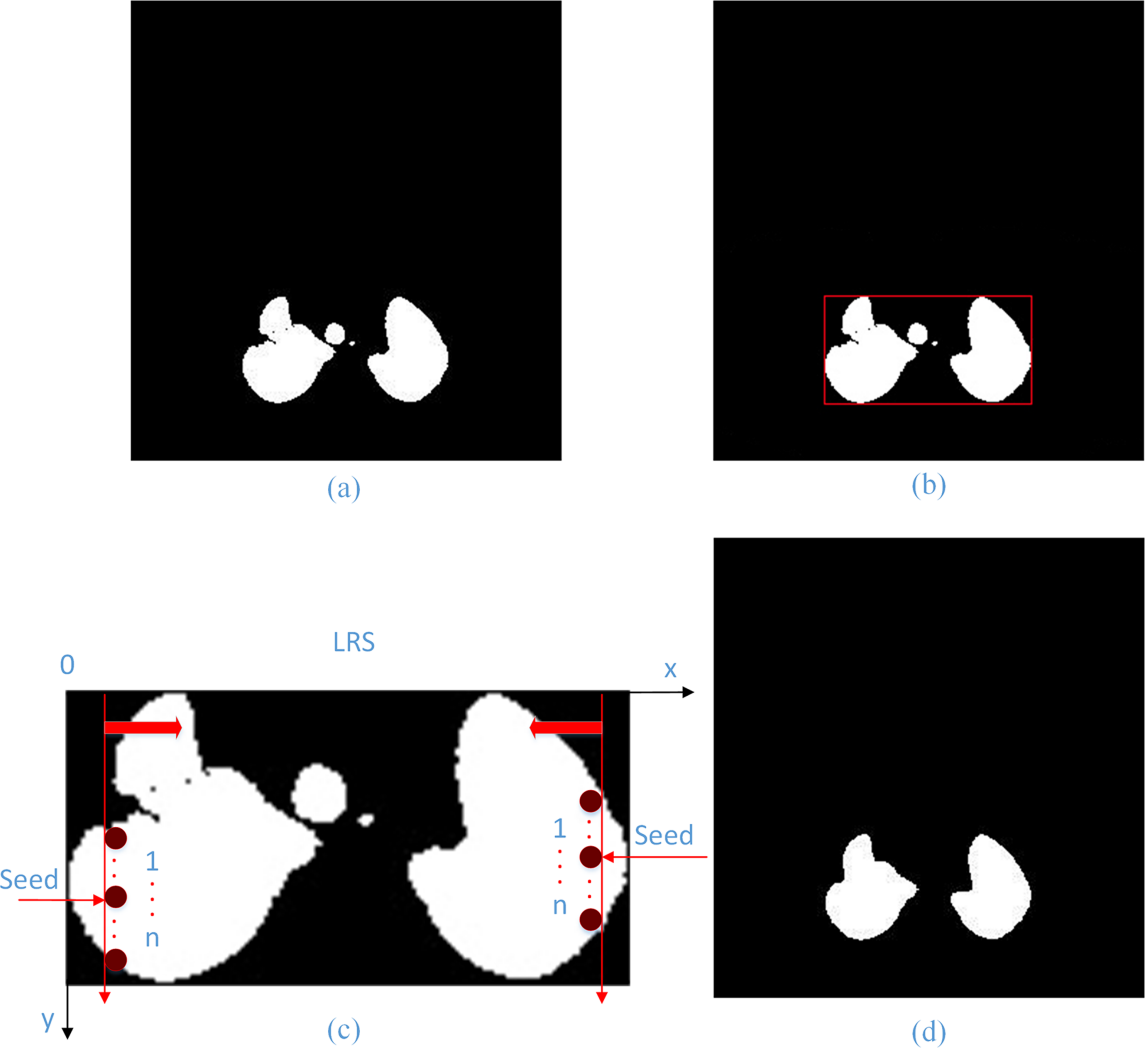
The process of trachea/bronchus removing and lung contour refining. (a) Binarization of the coarse lung image; (b) Extraction of minimum bounding rectangle; (c) Select seed points with the LRS algorithm; (d) Final lung mask.

We first use adaptive threshold method for image binarization and extract the minimum bounding rectangle of the lung. And then LRS algorithm is employed to select left and right seed points. In LRS, scan the minimum bounding rectangle image along the left and right sides simultaneously until there are more than ***5*** consecutive points on ***y*** direction with the pixel value 255, and record the middle (third) one’s ordinate value as the seeds. Next we adopt the improved region growing method based on these seeds to discard disconnected trachea, bronchus and other noise and extract lung out. Finally, dilation and erosion are used to smooth the contour and eliminate some vessels, small nodules as well as bones. Thus we acquire the final lung mask sequences with which to segment lung parenchyma image sequences accurately. The process of removing the trachea/bronchus and refining the lung contour is shown in **Fig 6.**

## Results

To verify our method’s validity and universality on lung parenchyma segmentation for these four types of lung CT image sequences, we compare the results of our method with those of some existing algorithms, such as the active contour model (**ACM**) [26], the watershed (**Watershed**) [27], region growing (**RG**) [28] and the level set (**Level Set**) [29], and with manual segmentation by two experts. All our implementations were programmed in the Microsoft Visual Studio 10.0 environment and executed on a personal computer equipped with a 3.40GHz Intel Core i7-3770 processor with 8 GB RAM. The software packages we used for medical image processing and 3D visualization are ITK 4.4.2 and VTK 6.1.0.

### 3.1 Qualitative evaluation

A solitary pulmonary nodule (SPN) is one of the most common types of pulmonary nodules. In this paper, for a series of lung CT image sequences with solitary pulmonary nodules, we use our algorithm and the ACM, watershed, RG and level set algorithms for lung segmentation. We must set up some of the necessary parameters to ensure the accuracy and effectiveness of the segmentation method. The values of these parameters are shown in **Table 5**.

**Table 5:** Parameter values setting.

Methods	Parameters	Values
Our method	***N***, ***n***, ***S***, *δ*, ***E*** in GSLIC	1000, 3, 16, 10, 0.0001
	***k***, ***p***, ***T***, *τ*, *η*, *μ* in SGNF	3, 6, 800, 0.7, 0.001, 0.0001
RG	***Threshold***	100–120
Watershed	***Level***	0.05, 0.1 and 0.15
ACM	***image spacing*, *expansion coefficient***	5, 2.0
Level set	***time threshold*, *stop time***	100,500

Because of the large number of image sequences, we select five lung CT images from the top to the bottom of the lung in a dataset with SPN and then use one image out of every twelve to demonstrate the process and the results of lung image segmentation.

**Fig 7:**
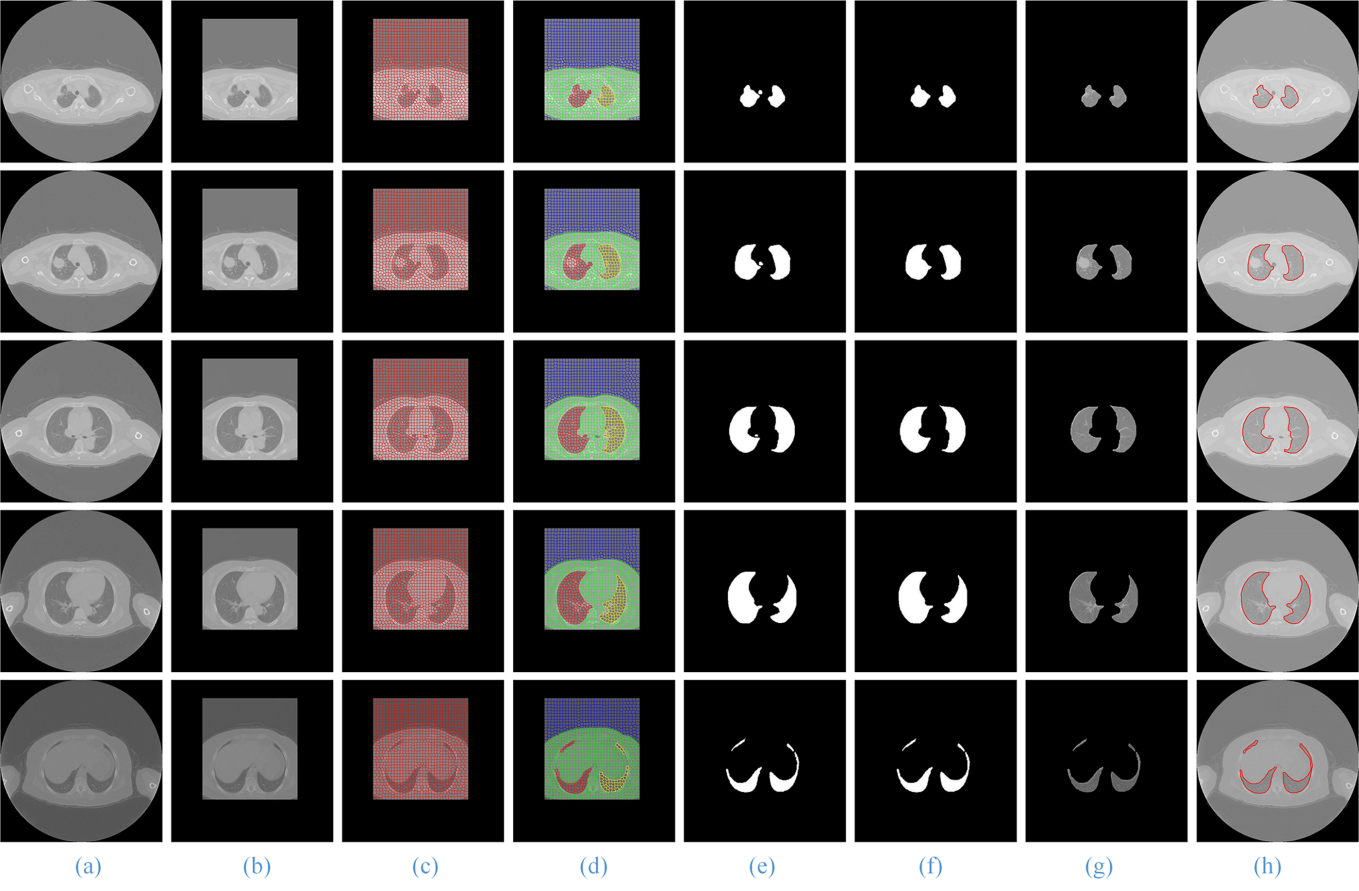
The segmentation results of our proposed method. Column (a) and (b) are five original lung CT images and ROI images from the top to the bottom; (c) and (d) shows the results of GSLIC and SGNF; (e) and (f) are the coarse and the final lung parenchyma mask; (g) and (h) present the final results of the proposed method and manual segmentation.

The process and results of our method are shown in **Fig 7**. Column (b) is the ROI extraction result of the original lung CT image sequences column (a); column (c) is the result using the SGLIC algorithm for superpixel segmentation; column (d) is the result of using the SGNF algorithm for clustering, which is optimized by the genetic algorithm; column (e) and (f) are the coarse and the final lung parenchyma mask; and columns (g) and (h) are the final segmentation results of our method and the artificial segmentation.

**Fig 8:**
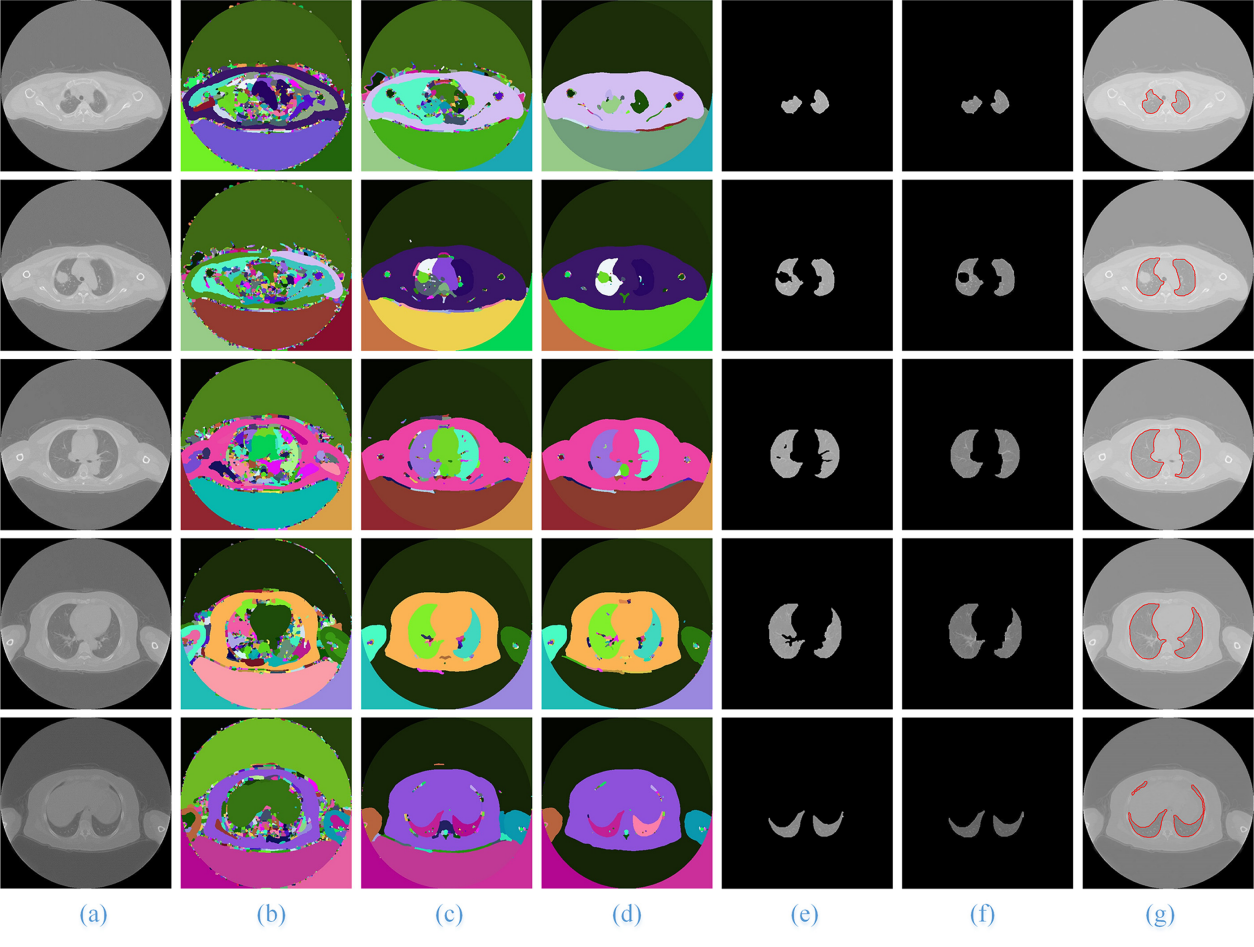
The segmentation results of using the watershed method. Column (a) is the five original lung CT images from top to bottom; (b)-(d) show the results of the level values of 0.05, 0.1 and 0.15, respectively; and (e) and (f) present the final results using the watershed algorithm and manual segmentation.

**Fig 9:**
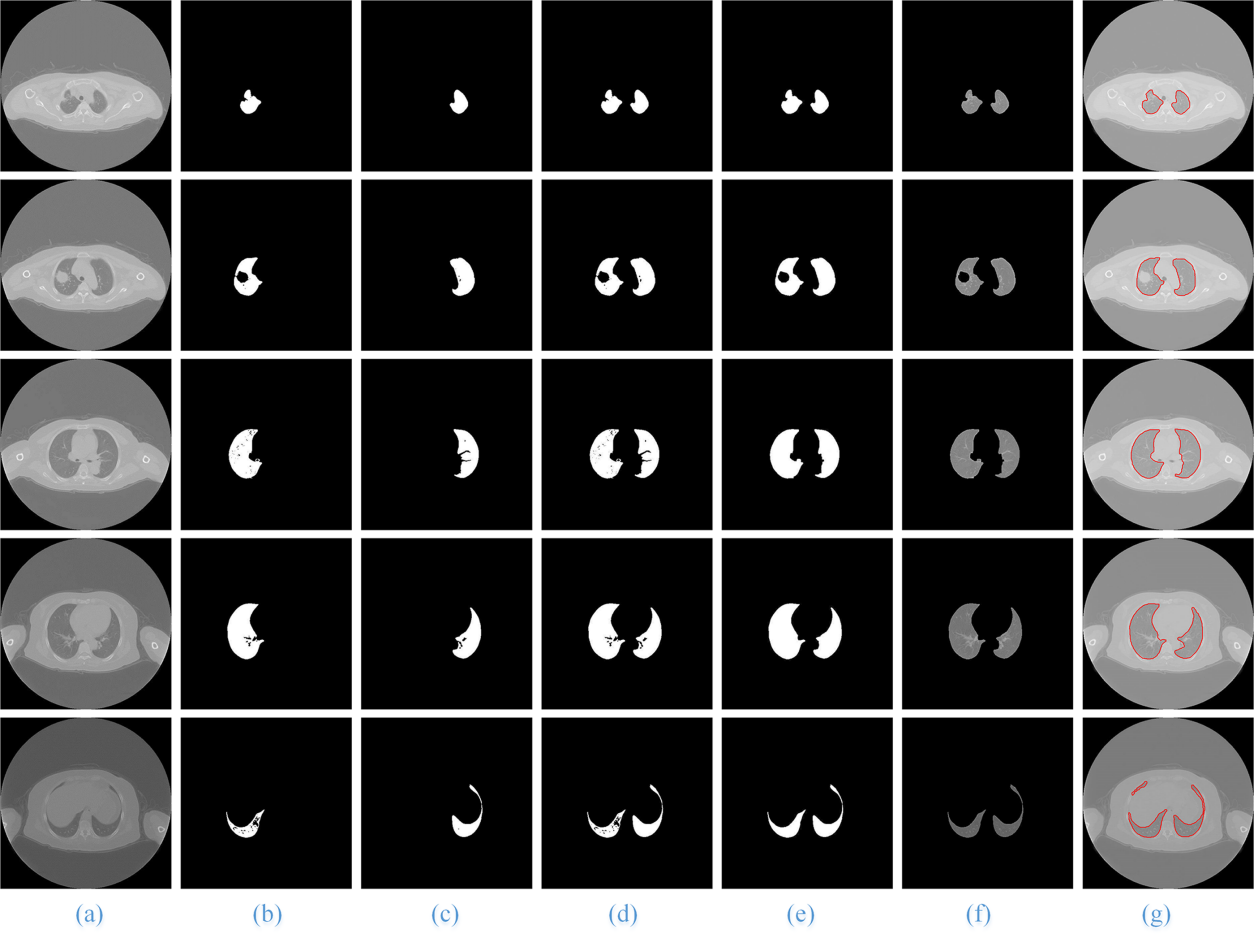
The segmentation results using the RG (region growing) method. Column (a) is the five original lung CT images from top to bottom; (b)-(d) show the mask of the left, right and whole lung; (e) are the coarse lung parenchyma mask; (f) and (g) present the final results of using RG algorithm and manual segmentation.

When using the RG, watershed and active contour model algorithms for sequence image segmentation, we set up the left and right lung seed points and select five lung CT images based on the result of the experiment. In this paper, the coordinates of these ***seed points*** from top to bottom are (235, 272) and (206, 276); (212, 277) and (331, 260); (187, 249) and (341, 251); (194, 245) and (343, 265); and (195, 304) and (333, 327), which will be used to segment the left and the right lung parenchyma images. The process and results of using the watershed and RG segmentation algorithms are shown in **Fig 8** and **Fig 9**. We have observed that when the watershed algorithm is used to segment the images, different ***level*** values will have different results (**Fig 8**, Column (b)—(d)). Compared with the segmentation results of the level values of 0.05, 0.1 and 0.15, the best level value of the best segmentation results is 0.15, and the coarse and final segmentation results are shown in **Fig 8**, Column(e) and (f). In addition, when using the RG algorithm, the ***threshold*** of the best segmentation result is between 100 and 120 (**Fig 9**, Column (f)).

**Fig 10:**
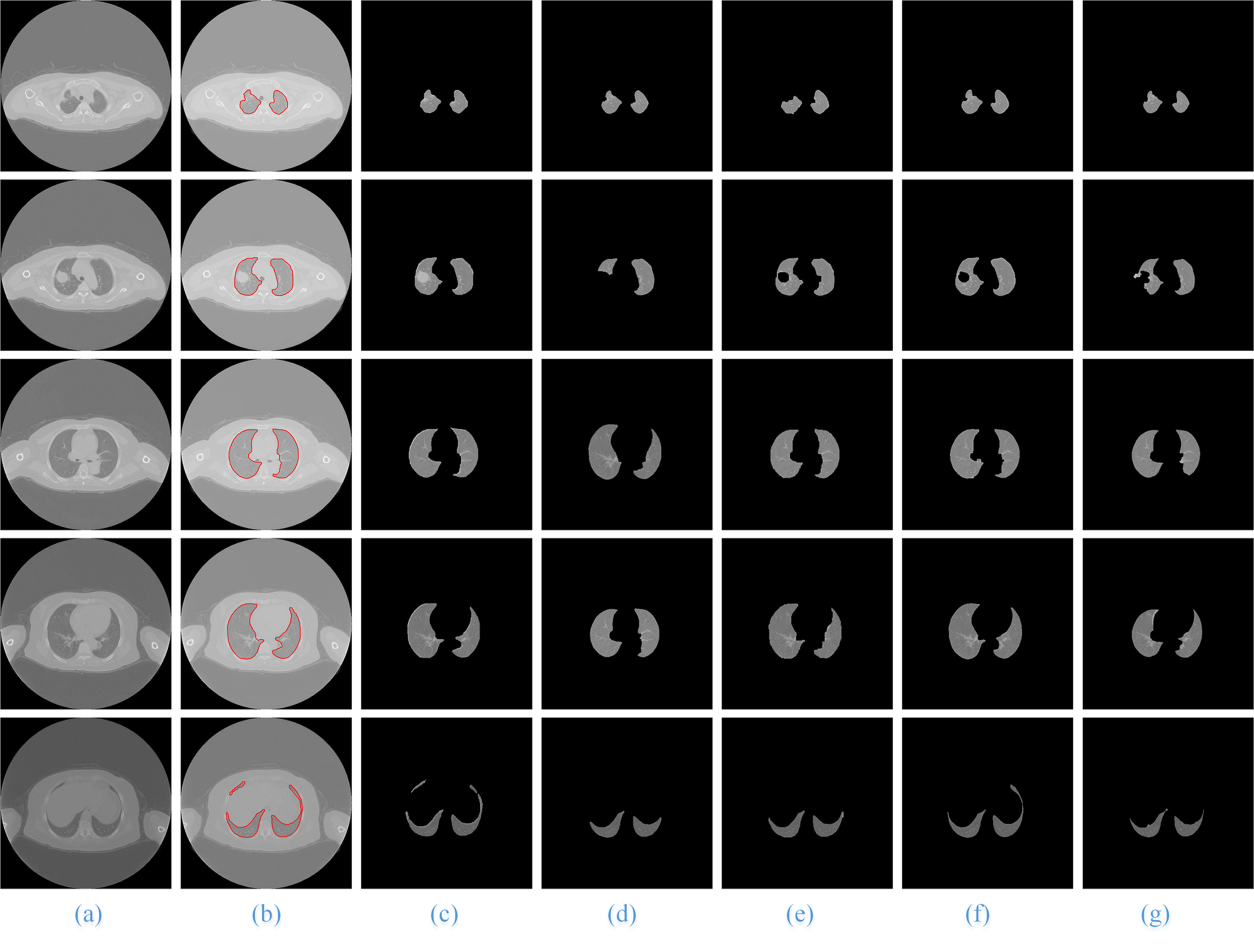
The comparison of the final segmentation results lung parenchyma scans with *malignant SPN*. Column (a) is the five original lung CT images from top to bottom, and (b)-(g) show manual segmentation; our proposed method; and the ACM, watershed, RG and level set methods, respectively.

**Fig 11:**
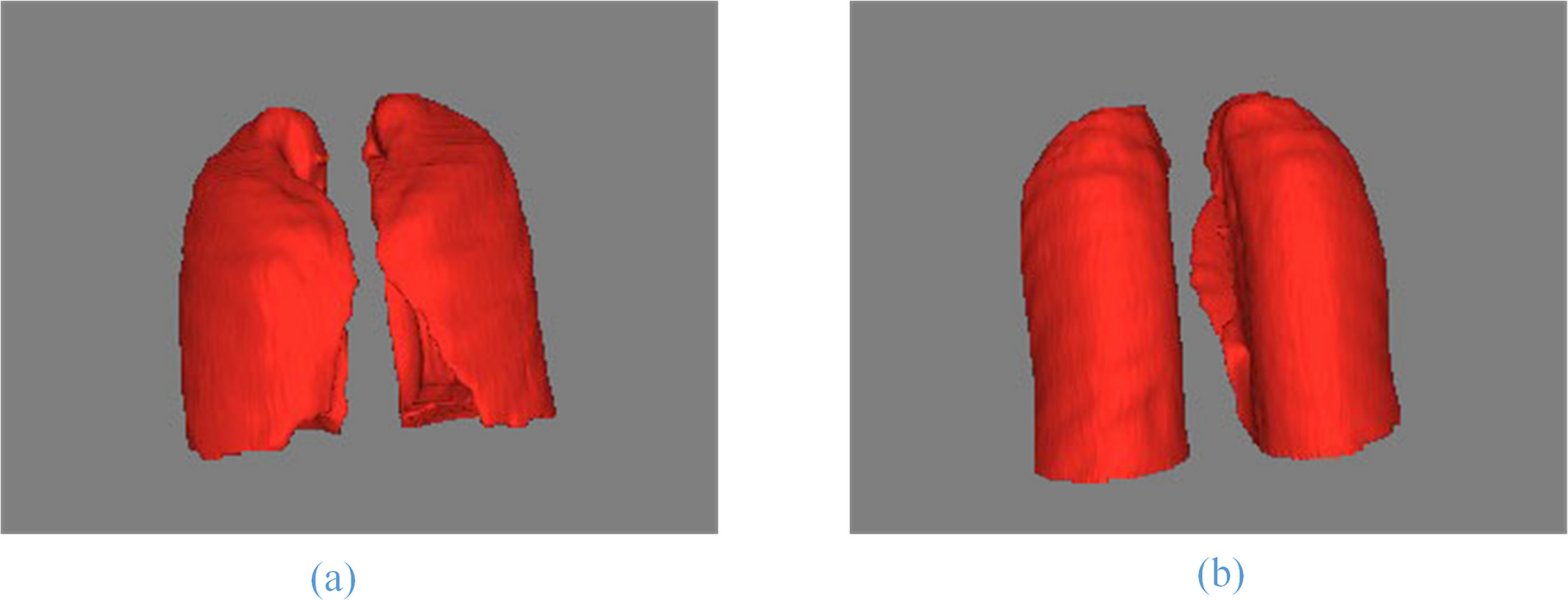
The results of the lung parenchyma image 3D reconstruction. (a) and (b) show the front and back of the whole lung.

In **Fig 10**, for the CT images with SPN, we give the comparison of the artificial segmentation results of the 5 methods. The best segmentation result is obtained by using the ACM to segment, the ***image spacing*** we select is 5 pixels, and the ***expansion coefficient*** value is 2.0 (**Fig 10**, Column (d)). When we use the level set algorithm for segmentation, the best segmentation result is obtained when the ***time threshold*** is 100 and the ***stop time*** is 500 (**Fig 10**, Column (g)). **Fig 11** shows the front and back of the lung in the 3D reconstruction of the lung parenchyma image sequences segmentation results with our proposed method using VTK.

**Fig 12:**
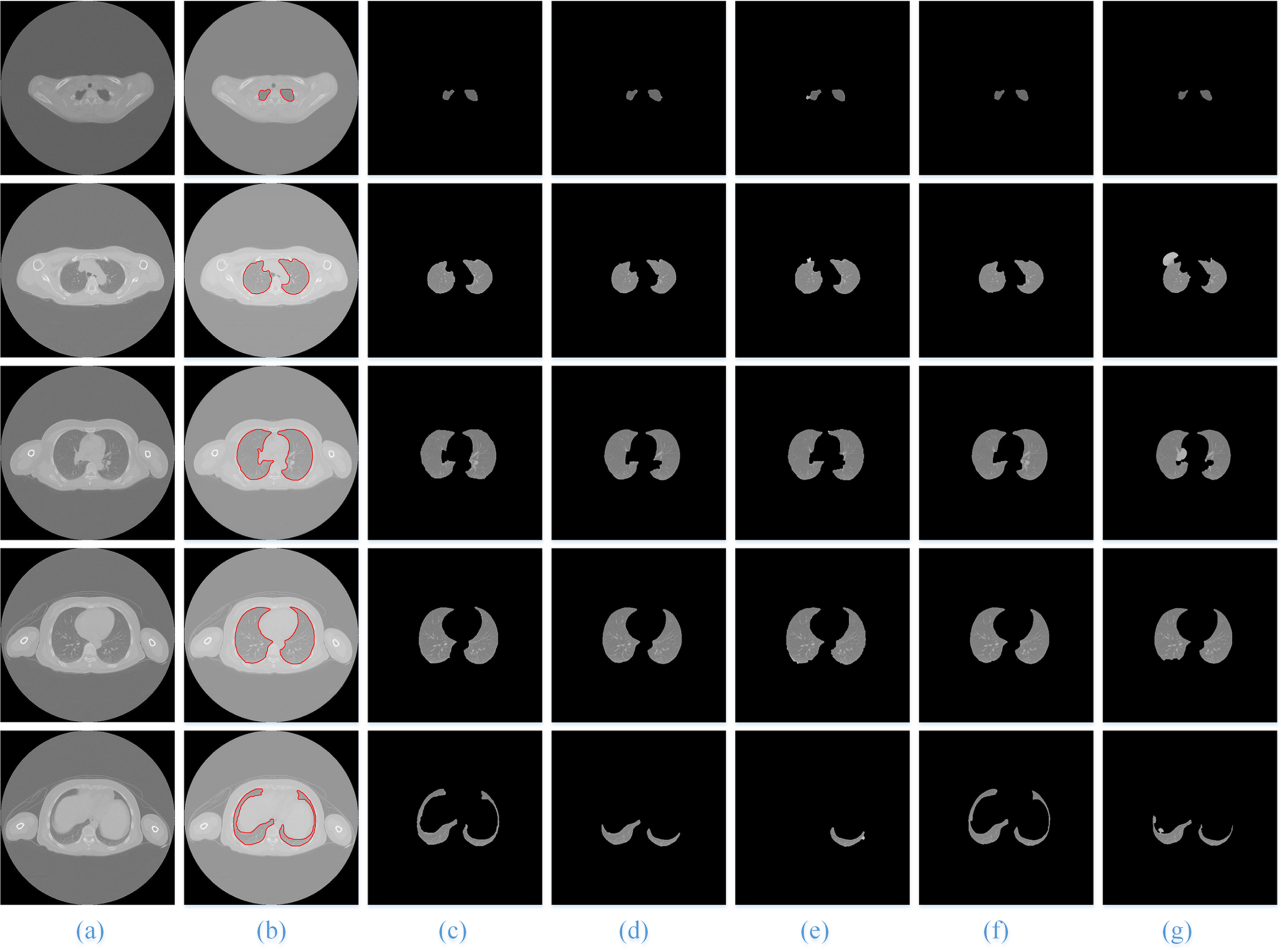
The comparison of the final segmentation results of lung parenchyma scans without nodules. Column (a) is the five original lung CT images from top to bottom, and (b)-(g) show manual human segmentation; our proposed method; and the ACM, watershed, RG and level set methods, respectively.

**Fig 13:**
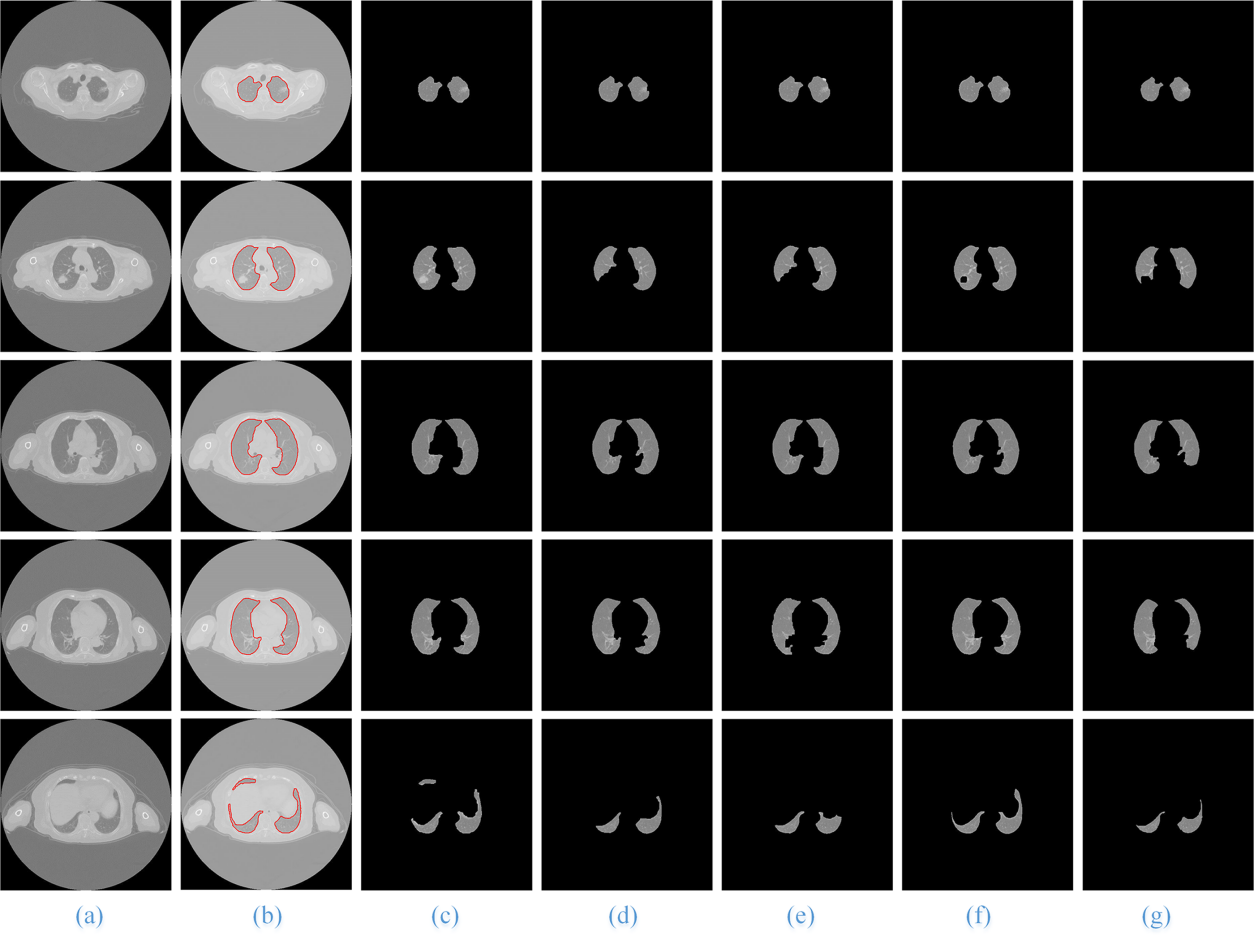
The comparison of the final segmentation results of lung parenchyma scans with benign nodules. Column (a) is the five original lung CT images from top to bottom, and (b)-(g) show manual human segmentation; our proposed method; and the ACM, watershed, RG and level set methods, respectively.

**Fig 14:**
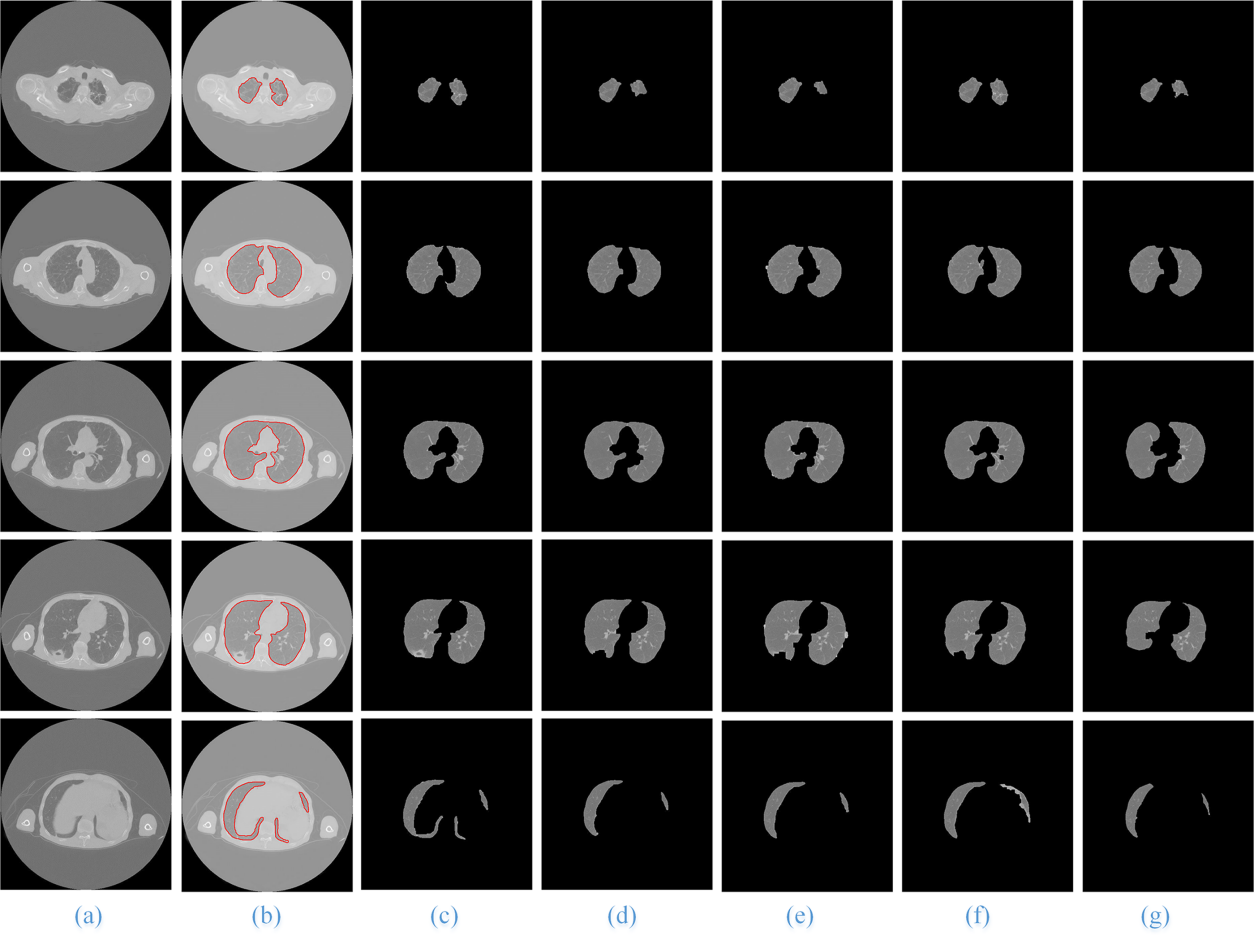
The comparison of the final segmentation results of lung parenchyma scans with pleural nodules. Column (a) is the five original lung CT images from top to bottom, and (b)-(g) show manual human segmentation; our proposed method; and the ACM, watershed, RG and level set methods, respectively.

For the other three types of lung sequence images, we also compare the segmentation results of these five segmentation methods without nodules (**Fig 12**), with benign nodules (**Fig 13**), and with pleural nodules (**Fig 14**).

When the experimental results are compared with the results of manual segmentation, it is shown that the method in this paper has the best segmentation results, particularly for the segmentation of irregular lung images, such as the top and the bottom of the lung and the pleural nodules in CT image sequences, and the advantages are more obvious.

For the lung sequences images without nodules (**Fig 12**), the segmentation results of our method (**Fig 12**, Column (c))and the region growing method (**Fig 12**, Column (f)) have good segmentation results, but the segmentation results of the watershed (**Fig 12**, Column (e)) and level set algorithms (**Fig 12**, Column (g)) are relatively poor.

For the lung image sequences with benign nodules (**Fig 13**, Row 2), the ACM and watershed algorithm will lose some of the lung parenchyma (**Fig 13**, Column (d) and (e)); the RG and level set algorithms will lose some of the pulmonary nodules (**Fig 13**, Column (f) and (g)); and the method in this paper has the best segmentation results (**Fig 13**, Column (c)).

For the lung sequence images with malignant SPN (**Fig 10**, Row 2), the algorithm in this paper can ensure the integrity of the segmentation of the lung parenchyma (**Fig 10**, Column (c)), while ACM will lose some of the lung parenchyma (**Fig 10**, Column (d)) and the watershed, RG and level set algorithms (**Fig 10**, Column (e)—(g)) will lose some of the pulmonary nodules.

For the lung sequence images with pleural nodules (**Fig 14**, Row 4), the ACM, watershed, RG and level set algorithms will miss the retraction part of the pulmonary and pleural nodules (**Fig 14**, Column (d)—(g)), while the method in this paper can guarantee much of the retraction part of the pulmonary and pleural nodules (**Fig 14**, Column (c)) and is the closest to the manual segmentation results (**Fig 14**, Column (b)).

In addition, for all CT images from the top to the bottom of the lung, the method in this paper is the only one that can ensure the integrity of the segmentation. Therefore, our proposed method has a better segmentation result and a higher generality on segmentation of lung parenchyma images.

### 3.2 Quantitative comparisons

Quantitative evaluation has significant importance in objectively assessing the effectiveness of an algorithm. The probabilistic rand index (PRI) [30], variation of information (VoI) [31] and *Jaccard* similarity coefficient [32] (Kim et al., 2005) are used to objectively assess the performance of the proposed algorithm.

Assuming that the original lung image *S* contains *M* pixels, the referential and actual segmentation results are expressed as ***S***_***s***_ and ***S***_*r*_, respectively, and the following conditions theoretically should be have met (12):

$$\overset{K}{\underset{k=1}{\cup }}S_{k}=\overset{N}{\underset{n=1}{\cup }}S_{n}=S$$ (12)

where *K* and *N* are the number of segmented regions in the referential and the actual segmentation results, respectively.

The probabilistic rand index (PRI) is a parameter to evaluate the consistency of attribute symbiosis between the actual segmentation results and the reference.

For a pixel pair (*x*_*i*_, *x*_*j*_) in the original lung image *S* marked (*s*_*i*_, *s*_*j*_) with the same attributes in the referential segmentation result ***S***_***s***_, which should be the same in ***S***_***r***_, the value of PRI [30] can be calculated as (13):

$$PRI(S_{s},S_{r})=\frac{1}{\left(\begin{array}{c} M\\ 2 \end{array}\right)}\sum_{i}\sum_{j(j\neq i)}\left(I(s_{i}=s_{j}\&\&r_{i}=r_{j})+I(s_{i}\neq s_{j}\&\&r_{i}\neq r_{j})\right)$$ (13)

where ***I*** is a discriminant function that is used to determine whether the pixel pair has the same label. The value of PRI is in the range of [0, 1], and the larger the value, the better the result.

The variation of information (VoI) [31] is a measure of information content that depicts how much one segmentation reflects the information of the other segmentation. It is the conditional entropy among the distributions of the segments labels. Therefore, the VOI value can be calculated as:

$$VoI(S_{s},S_{r})=H(S_{s})+H(S_{r})-2I(S_{s},S_{r})$$ (14)

where *H(S*_*s*_*)* and *H(S*_*r*_*)* represent the entropy, and *I(S*_*s*_, *S*_*r*_*)* represents the mutual information. *H(S*_*s*_*)* and *I(S*_*s*_, *S*_*r*_*)* also can be calculated as (15) (16) and (17).

$$H(S_{s})=\text{-}\sum_{k=1}^{K}P(k)\log P(k)$$ (15)

$$H(S_{r})=\text{-}\sum_{k=1}^{K}P(n)\log P(n)$$ (16)

$$I(S_{s},S_{r})=\sum_{k=1}^{K}\sum_{n=1}^{N}P(k,n)\log\frac{P(k,n)}{P(k)\;*\;P(n)}$$ (17)

The VoI values lie in [0, ∞). The 0 indicates that the two segmentations match perfectly. The smaller the value of VoI, the less information changes and the better the results will be.

The Jaccard similarity coefficient (Jaccard) is a measure to compare the similarity between the sample sets, which can indicate the coincidence degree of two images. The value of Jaccard can be calculated as (18).

$$Jaccard=\frac{M\left(S_{s}\;\text{I}\;S_{r}\right)}{M\left(S_{s}\;\text{U}\;S_{r}\right)}$$ (18)

The Jaccard values lie in [0, 1], and a higher Jaccard similarity coefficient indicates a better segmentation result.

**Table 6** shows the average scores of the PRI, VoI and Jaccard measures for the five algorithms on four types of lung CT image sequences. It is clear from **Table 6** that the proposed method outperforms the other state-of-the-art algorithms in terms of PRI, VoI and Jaccard.

**Table 6:** Average values of PRI, VOI and Jaccard for the five algorithms on four types of image sequences.

Types	Measures	ACM	Watershed	RG	Level set	GSLIC-SGNF
***Without-***	***PRI***	0.9581	0.9486	0.9611	0.9474	0.9624
***nodules***	***VoI***	1.779	2.283	1.754	1.4535	1.3815
	***Jaccard***	0.9375	0.9252	0.9385	0.9267	0.941
***Benign-***	***PRI***	0.8624	0.8964	0.9285	0.8695	0.9465
***nodules***	***VoI***	1.9532	2.369	2.7471	1.8382	1.6515
	***Jaccard***	0.8845	0.8972	0.9238	0.8758	0.9342
***Malignant-***	***PRI***	0.8948	0.9025	0.9094	0.8989	0.9169
***SPN***	***VoI***	1.9875	2.6475	2.8305	1.8975	1.8195
	***Jaccard***	0.8708	0.8636	0.902	0.8361	0.9257
	***PRI***	0.8556	0.8629	0.8505	0.8636	0.8926
***Pleural-***	***VoI***	3.825	3.323	4.152	2.8136	2.2052
***nodules***	***Jaccard***	0.8122	0.8535	0.7934	0.8242	0.8821

For the lung sequences images of without nodules, five methods have better segmentation results. The average PRI and Jaccard values of the RG algorithm are close to the proposed method but the average VoI values are much greater. The watershed algorithm has a minimum PRI value of 0.9474, while the level set algorithm has a maximum VoI value and the lowest Jaccard value. Moreover, for lung sequence images with benign nodules, malignant SPN and pleural nodules, the RG algorithm’s performance drops rapidly with the highest VoI value, while the watershed and level set algorithms are relatively stable. Our method has better results in term of PRI, VoI and Jaccard values.

In general, our proposed method has the best segmentation performance in terms of the parameter comparison of the five types of image segmentation. From lungs without nodules to lungs with pleural nodules, our method’s performance declines slightly. The main cause perhaps is that the lung parenchyma images contain lung nodules, and the types of lung nodules are becoming more and more complicated. It is clear from **Table 6** that the RG algorithm is the most sensitive to lung nodules. Unless otherwise stipulated in the image sequences without nodules, the RG algorithm is very close to the proposed method, but once lung nodules are included, there is a drastic decline in the indicators.

**Fig 15:**
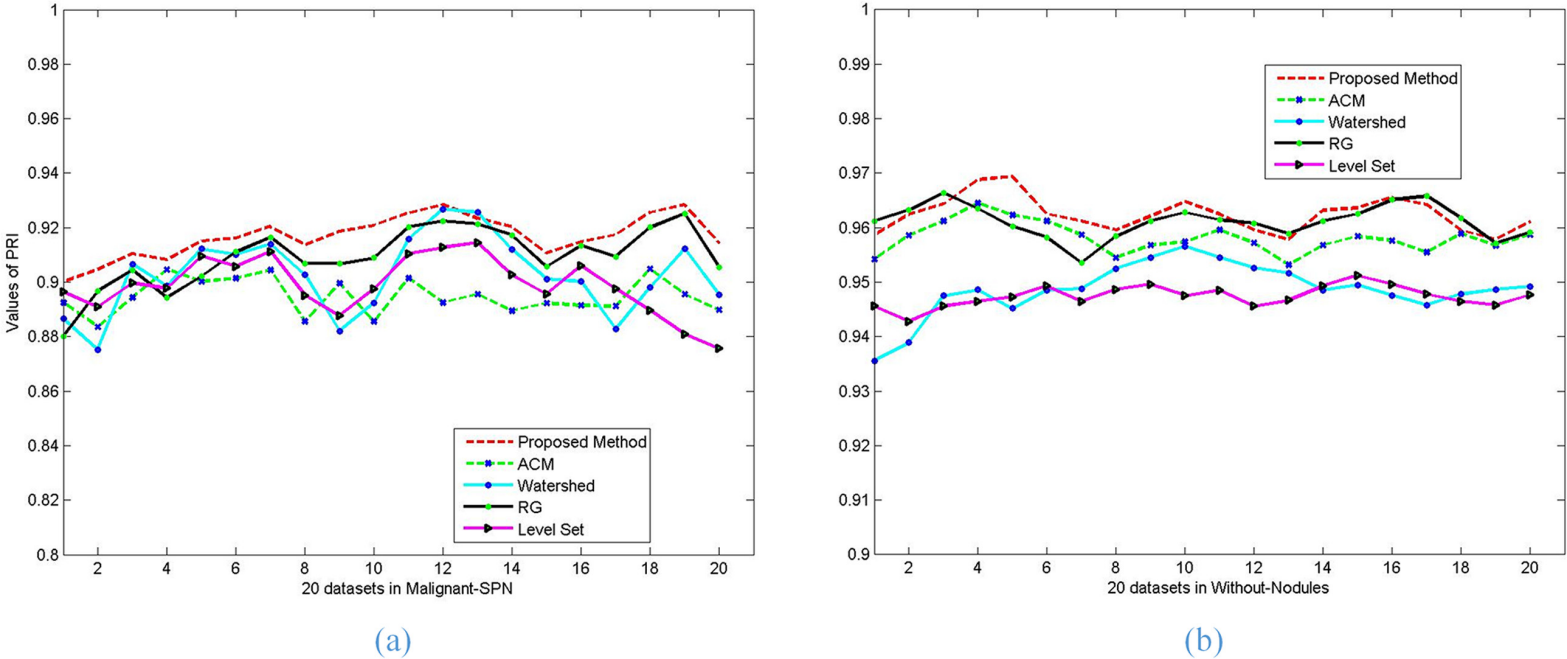
The *PRI* values of the five methods on the segmentation results of lung parenchyma scans with *malignant SPN* (a) and *without nodules* (b).

**Fig 16:**
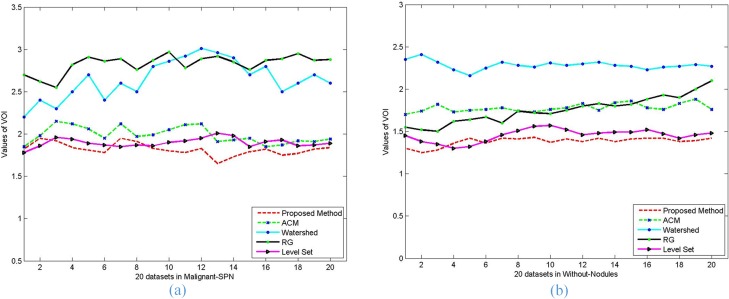
The *VOI* values of the five methods on the segmentation results of lung parenchyma scans with *malignant SPN* (a) and *without nodules* (b).

**Fig 17:**
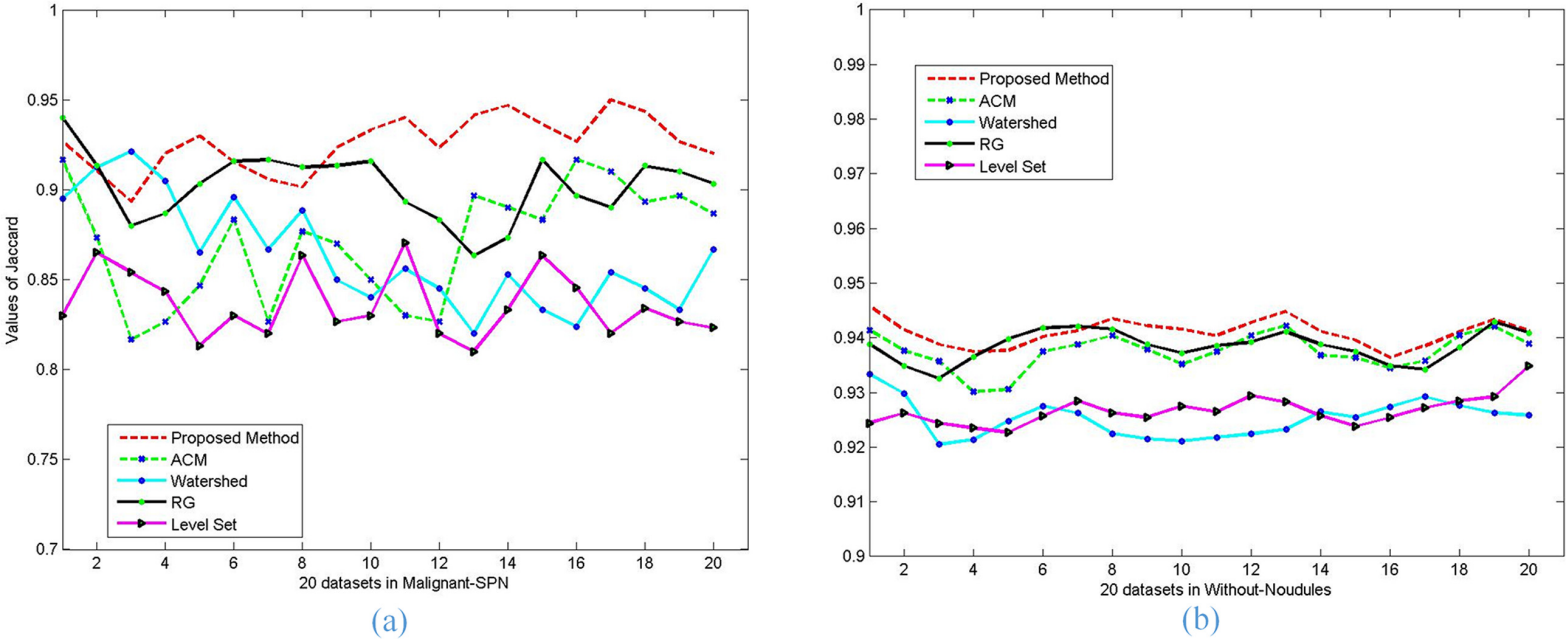
The *Jaccard* values of the five methods on the segmentation results of lung parenchyma scans with *malignant SPN* (a) and *without nodules* (b).

Moreover, the performance of each of the five algorithms in terms of PRI, VoI and Jaccard values on each image with malignant SPN (a) and without nodules (b) is graphically represented in **Fig 15**, **Fig 16** and **Fig 17**. It is clear from **Fig 15** that the proposed method performs better than the other methods in terms of the PRI value, the RG algorithm is close to the proposed method and better than the other algorithms. **Fig 16** shows that our proposed method can always keep the global minimum value of VOI, while the level set value nearly approaches ours. Both the Watershed and RG algorithm have higher VoI values. It is obvious that our Jaccard value is higher than that of RG and is far better than for the other methods.

In consequence, based on the comparison and analysis of the three measures PRI, VoI and Jaccard, our method is the most close to the artificial segmentation results, which can further reflect our method’s high preformation and wide generality in the segmentation of lung parenchyma images.

**Fig 18:**
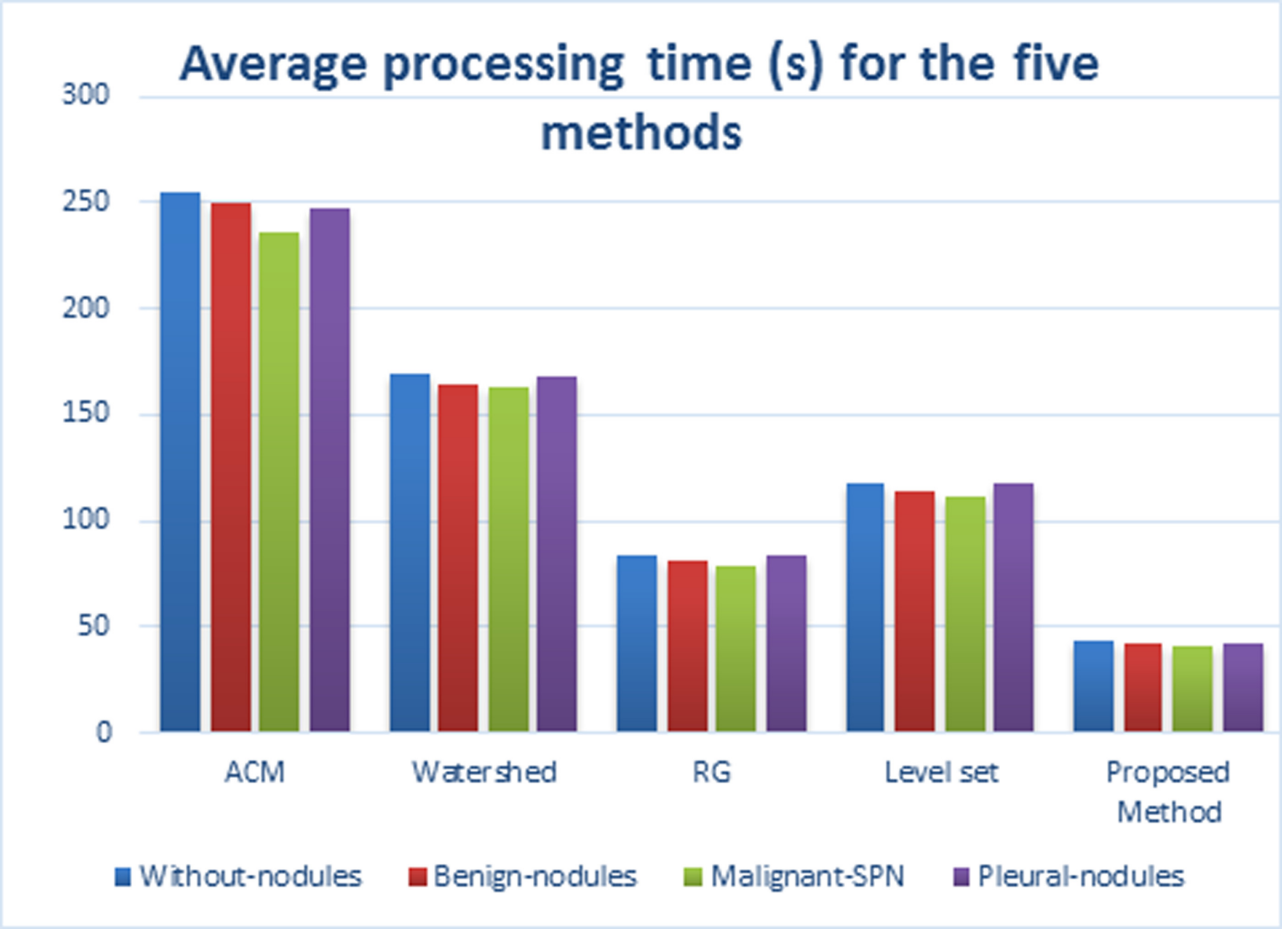
The average processing time of the five methods on the segmentation results of lung parenchyma scans.

For the four types of lung parenchyma images, we also analyzed the time performance of the five methods, as shown in **Table 7**, and the average processing times for the five methods are shown in **Fig 18**. The “Average Dataset size” row signifies the average number of lung CT images in a dataset. It is clear from **Fig 18** that the average processing time for each dataset using our method is 42.21 seconds; i.e., it will take 0.71 seconds to process a single slice, which is far better than in other four methods. Therefore, the proposed method has obvious advantages over the other methods in terms of segmentation speed of lung CT images.

**Table 7:** Average processing time(s) for the five algorithms on CT image sequences.

Types	Average Dataset size	ACM	Watershed	RG	Level set	GSLIC-SGNF
Without nodules	512*512*60	254.57	169.26	83.35	117.56	43.22
Benign nodules	512*512*59	250.42	164.45	81.22	114.28	41.69
Malignant SPN	512*512*60	235.51	162.88	78.92	111.36	41.18
Pleural nodules	512*512*62	246.84	168.23	83.25	118.23	42.76

## Conclusion

Our work indicates that our proposed method can segment various types of lung parenchyma image sequences effectively. This method is more accurate and universally applicable than any of the traditional methods. Based on the segmentation of the four different types of sequences of lung CT images, which included 4812 images from 80 datasets, we compare the results of our method and those of the existing algorithms with manual segmentation. The experimental results show that our method can achieve accurate segmentation of the lung parenchyma and in particular accurate segment the lung CT images, which have complex morphological structures such as the top and bottom of the lung and contain pulmonary nodules. Our method can achieve an average volume pixel overlap ratio of 92.22 ± 4.02% for the four types of lung parenchyma image sequences. Moreover, our method is less time consuming, with an average processing time of 42.21 seconds for each dataset, meaning it takes approximately 0.71 seconds to process a single slice. Therefore, in the segmentation of lung parenchyma image sequences, taking the high correlation between adjacent slices of CT image sequences into consideration can significantly improve the speed of segmentation, while superpixels can guarantee the quality and the post-processing of image segmentation, and the SGNF optimized by the GA can be more effective at maintaining the integrity of the lung parenchyma segmentation.
